# TB or Not TB: Lung Nocardiosis, a Tuberculosis Mimicker

**DOI:** 10.7759/cureus.55412

**Published:** 2024-03-02

**Authors:** Laura M Gonzalez, Raksha Venkatesan, Pablo Amador, Raghavendra R Sanivarapu, Barath Rangaswamy

**Affiliations:** 1 Internal Medicine, Texas Tech University Health Sciences Center, Odessa, USA; 2 Pulmonary and Critical Care Medicine, Texas Tech University Health Sciences Center, Odessa, USA; 3 Pulmonary and Critical Care Medicine, Nassau University Medical Center, East Meadow, USA

**Keywords:** necrotizing pneumonia, nocardia species, nocardia in immunocompetent, pulmonary cavitation, lung nocardiosis

## Abstract

*Nocardia*, a gram-positive bacterium found in soil and water, rarely causes infections in immunocompetent patients. Diagnosing and treating nocardiosis can be challenging due to its infrequency and the similarity of its symptoms to other diseases. We describe the case of a middle-aged male with a history of latent tuberculosis who presented with hemoptysis. Imaging revealed a persistent lung mass, and pathology and microbiology studies confirmed *Nocardia* infection. The patient was treated with antibiotics and discharged home. Pulmonary nocardiosis can mimic tuberculosis, fungal infections, or malignancies. Immunocompetent patients make up one-third of the cases. Diagnosis can be difficult, as the organism takes time to grow in culture, but molecular techniques and histology can aid in diagnosis. Treatment often involves a six- to 12-month course of trimethoprim-sulfamethoxazole (TMP-SMX). Prompt identification of the etiological agent is essential for effective treatment, especially for immunocompetent patients who may not exhibit typical risk factors.

## Introduction

*Nocardia* is a type of bacteria belonging to the aerobic gram-positive, branching actinomycetes group. It is commonly found in soil and water [1]. This bacterium is believed to affect immunocompromised individuals primarily [2]. In the United States, it is estimated that 500-1,000 new cases of nocardiosis infections are reported each year [3]. Due to the relative rarity of nocardiosis and the fact that its symptoms can resemble those of other diseases, diagnosing and treating this infection can be challenging [4,5]. Treatments can be cumbersome, requiring a prolonged course of antibiotics. However, selecting the most effective antibiotic regimen can be challenging due to the variable susceptibility of *Nocardia* to different antibiotics. Treatment may involve a combination of antibiotics tailored to the specific strain identified, and the duration of treatment is often extended to prevent relapse [1,4,6]. Due to the rarity of nocardiosis, its potential to mimic other diseases, and its variable response to antibiotics, diagnosing and effectively treating this infection can pose a significant challenge for healthcare professionals.

## Case presentation

**Figure 1:**
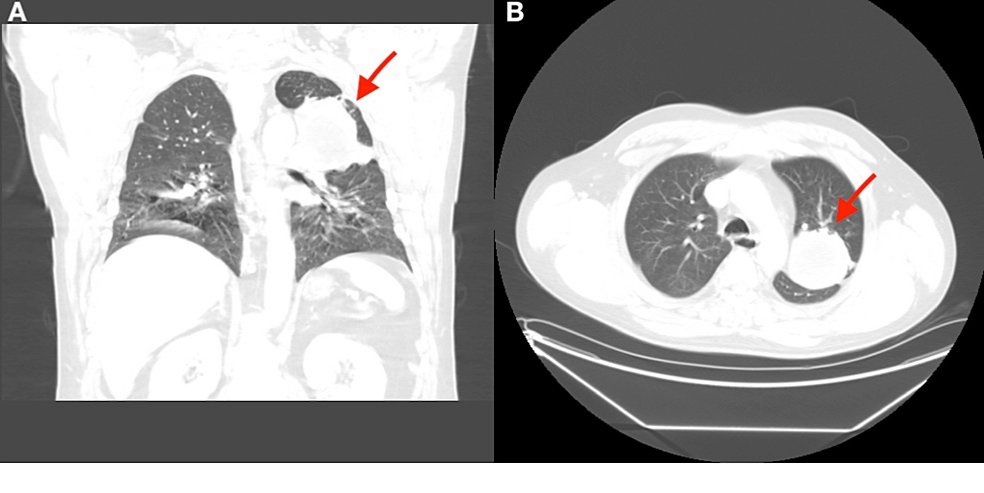
Computed tomography of the chest in coronal (A) and axial (B) views. It shows a left upper lobe mass (red arrow) which measures up to 6.7 cm in diameter. It contains a fluid density material and heterogeneously enhances.

A 56-year-old male, who is a former smoker, presented with recurrent hemoptysis and a productive cough for the last three days. He denied any chest pain, shortness of breath, fever, weight loss, or history of recent travel. He has a past medical history of latent tuberculosis that was partially treated. He is also known to have had a left upper lobe cavitary mass for the past 10 years, accidentally discovered when he was being treated for a left clavicular fracture. X-rays and computed tomography (CT) of the chest during that time showed a mass with a fluid density in the left upper lobe measuring 6.7 cm in diameter (Figure 1).

Three years prior to the current presentation, he was admitted for hemoptysis and underwent fiberoptic bronchoscopy, which only showed a necrotic-appearing mass-like lesion completely obstructing the apical posterior segment of the left upper lobe. The biopsies taken during this procedure demonstrated benign lower respiratory mucosa with marked acute inflammation and bacteria. Microbiology tests returned positive for branching gram-positive bacilli; consequently, he was treated for nocardiosis. He was later hospitalized with the same chief complaint of hemoptysis (six months prior to the current presentation). At the time, the infectious work-up was negative, and he had three negative sputum results for acid-fast bacilli. He was discharged home on voriconazole because of the possibility of fungal infection, and he was offered surgical intervention, which he declined.

On this admission, his vitals were as follows: oxygen saturation of 96% on room air. His heart rate was 65 bpm, and he was afebrile (36.7°C) and normotensive (130/62 mmHg). The patient was alert and oriented to time, place, and self. The chest was symmetrical, and the trachea was at the midline. Air entry was good bilaterally, with bronchial breath sounds involving the left upper lung, but otherwise, there was no obvious wheezing or rhonchi. Heart sounds were regular and no added murmurs were heard. 

Laboratory results on admission did not show any outstanding findings. There was no leukocytosis (10.190x103/uL); however, neutrophils were elevated on the differential (73.5%). He was found to have mild chronic normochromic normocytic anemia (hemoglobin, 12.8 g/dL; mean corpuscular volume (MCV), 81.9 fL; mean corpuscular hemoglobin (MCH), 28.6 pg), and platelets were within normal limits (361x103/uL). The comprehensive metabolic panel was within normal limits. 

**Figure 2:**
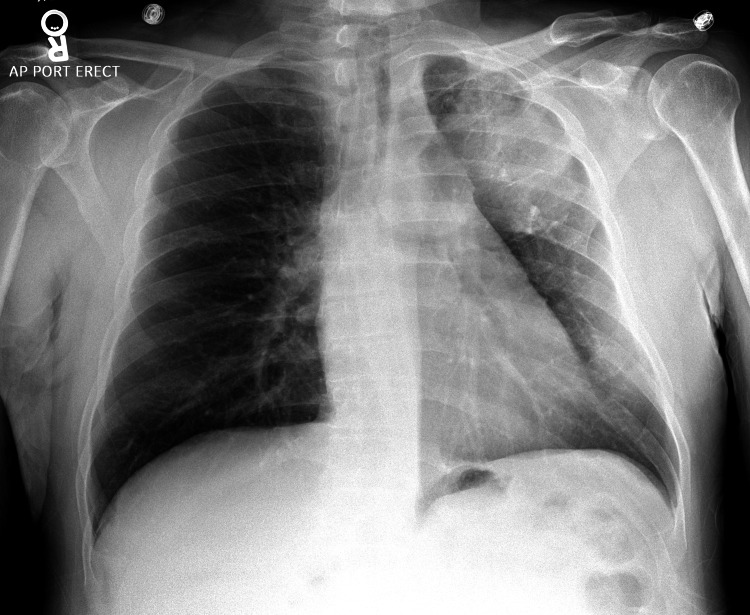
Chest X-ray in the anteroposterior view done on the day of current admission. There is a large mass in the left upper lung, which appears to have progressed slightly. The right lung is clear. There is no evidence of pneumothorax or pleural effusion. Also, it shows a remote left clavicular fracture.

**Figure 3:**
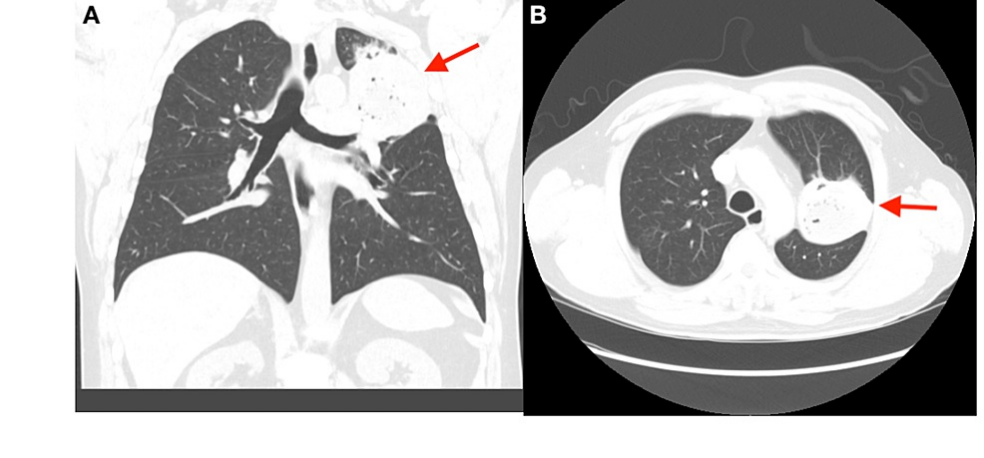
Computed tomography of the chest in coronal (A) and axial (B) views performed in current admission. It shows the same upper lung mass, which increased in size (red arrow). The mass currently measures 7.7 cm with a mild patchy infiltrate seen around the mass.

An initial chest X-ray revealed a persistent left upper lobe cavitary mass (Figure 2). A CT of the chest redemonstrated a cavitary lesion, which now measured 7.7 cm, with a mild patchy infiltrate seen around the mass (Figure 3). He was started on empiric antibiotics with doxycycline, ceftriaxone, and fluconazole for fungal coverage. 

Differential diagnosis

Despite the patient experiencing chronic symptoms and no evidence of metastasis after many years, it was imperative to rule out the possibility of malignancy. The patient had undergone bronchoscopy with bronchoalveolar lavage during a previous admission, which did not reveal any malignant cells. Luckily, tissue samples obtained after surgery confirmed that the cause was an inflammatory condition rather than a malignancy. Pathology findings indicated acute necrotizing cavitary granulomatous inflammation. Vasculitis such as granulomatosis with polyangiitis could present with this pathology. However, the patient did not present with involvement of other organs, such as glomerulonephritis, upper respiratory tract involvement, or multiple mononeuropathy. When a patient presents with chronic symptoms, such as our patient, it is crucial to consider *Mycobacterium tuberculosis*. However, our patient had previously received treatment, which he did not complete due to a lack of follow-up. Three consecutive acid-fast bacteria sputum stains were negative, indicating a negative tuberculosis result. Additionally, fungal infections were high on our list of differentials, but fungal cultures and silver stains were negative. *Aspergillus fumigatus* antibody IgG and galactomannan were negative, as well as *Coccidioides* antibodies and *Histoplasma* antibodies.

Treatment and outcome

**Figure 4:**
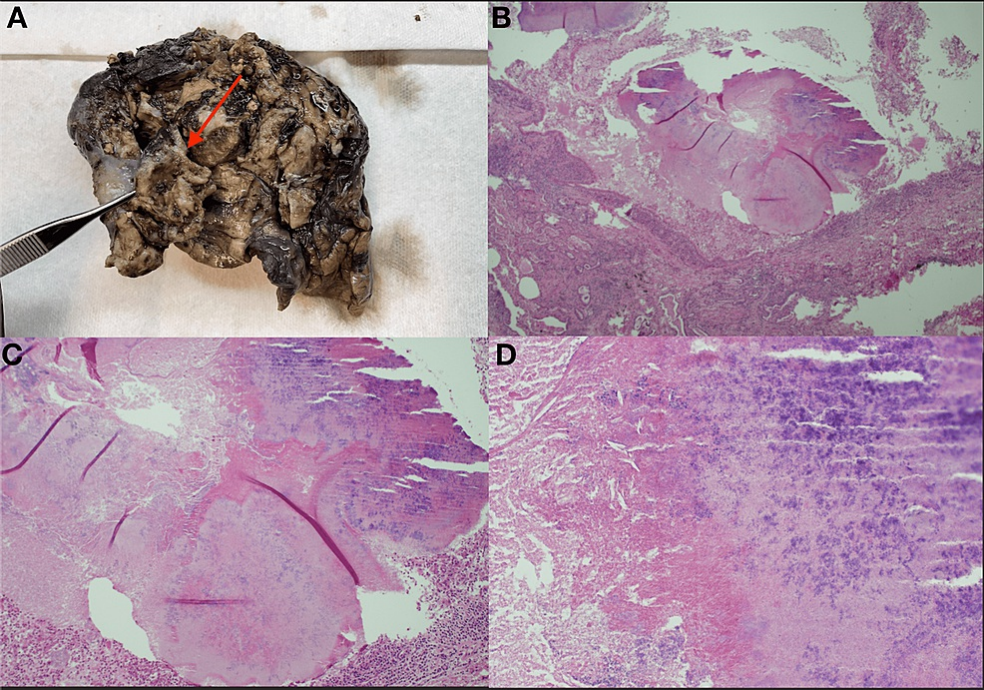
Pathology of the resected cavitary lesion in the left upper lobe. Picture A includes a macroscopic pathologic sample consisting of a lobectomy measuring 16x8x6.5 cm. There is a mass measuring 11 cm (red arrow) located near the pleura of the lung. This mass is located 3 cm from the bronchial margin. Dissection of the mass reveals a cavitary lung lesion filled with degenerative yellow-tan debris. Pictures B, C, and D correspond to low-, medium-, and high-power fields in the microscope of the mass. These show acute necrotizing cavitary granulomatous inflammation with chronic bronchitis.

**Figure 5:**
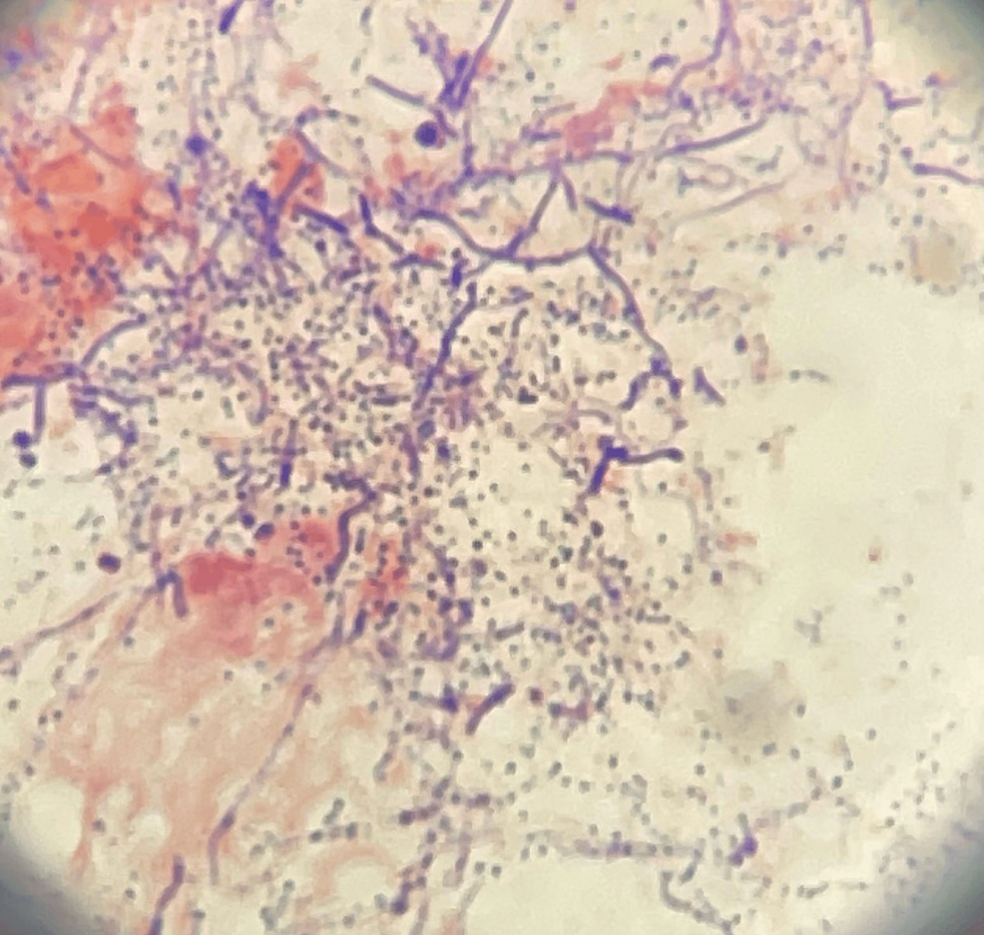
Microscopic view of the patient's tissue culture findings.

Given a persistent left upper lobe cavitation, the cardiothoracic surgeon was consulted, who recommended surgical intervention. Seven days later, the patient underwent a left thoracotomy with left upper lobectomy and intercostal nerve block. During the surgical procedure, a left upper lobe mass with atelectasis was found with dense pleural adhesions at the level of the apex and no evident pathologic lymphadenopathy. The patient underwent the procedure without complications. The pathology of the upper lobe is described as acute necrotizing cavitary granulomatous inflammation with chronic bronchitis (Figure 4). Microbiology studies demonstrated branching gram-positive bacilli, identified as *Nocardia* species, which was the same organism isolated three years prior during a bronchoscopy (Figure 5), confirming recurrence of pulmonary nocardiosis. The day after surgery, once these results returned positive, he was treated with intravenous meropenem and trimethoprim-sulfamethoxazole (TMP-SMX) for seven days. 

The postoperative state was complicated by persistent pneumothorax. For this reason, the patient was discharged home 11 days after the left upper lobectomy with a Heimlich valve and drainage bag with a hole cut for air passage. He was to continue long-term oral antibiotic therapy with amoxicillin-clavulanate and TMP-SMX for a total of one year and to follow up with the infectious disease specialist. After nine days from discharge, the chest tube was removed successfully with the resolution of pneumothorax.

## Discussion

*Nocardia* is an aerobic gram-positive rod and catalase-positive bacterium [1]. It is partially acid-fast and has filamentous hyphae-like branching bacteria within the *Actinomycetales* order [6,7]. Currently, there are 119 identified species of *Nocardia* as of 2020 [6]. This bacterium is commonly found in soil, decomposing vegetation, as well as fresh and saltwater environments [1,6]. Various factors can lead to the entry of *Nocardia* into the body, with aerosol exposure being the primary route [8]. Other causes include penetrating injuries, abrasions or cuts, gardening, insect bites, iatrogenic injections or procedures, exposure to contaminated water, near-drowning, and ingestion [6].

*Nocardia* spp. virulence is attributed to its ability to thrive and multiply in various human cells and avoid host immune response through the production of catalase and superoxide dismutase, inhibition of phagosome-lysosome fusion, reduction of intracellular acid phosphatase levels in macrophages, and secretion of toxins and hemolysin [1,4]. Furthermore, *Nocardia* can survive as facultative intracellular parasites within macrophages [1]. Recent data show that the production of tumor necrosis factor (TNF)-alpha after the stimulation of blood monocytes is important for the innate immune response to cell wall monomers of *Nocardia* spp. This is compatible with reports of cases of nocardiosis after treatment with TNF antagonists [9]. The crucial role of cell-mediated immunity has been proved in experimental in vitro studies, explaining why *Nocardia* sp. behaves as an opportunist microorganism in an immunocompromised host [10].

According to certain studies, pulmonary disease makes up around 80-85% of the cases [2,6]. Disseminated disease is responsible for 30% [11], while central nervous system involvement can be observed in 3-26% of individuals with nocardiosis [12]. Between 1950 and 1991, one-third of the 1000 cases of *Nocardia* occurred in patients with no identifiable underlying risk factors [1]. Some other reports suggest that even a higher percentage of cases (50%) occur in immunocompetent patients. The cases of *N. beijingensis* reported to date favor immunocompetent individuals [13]. 

The infection is prevalent among individuals with compromised cellular immunity, including those with HIV infection, long-term use of corticosteroids, malignancy, chronic alcoholism, diabetes mellitus, and a history of organ transplantation [5,6]. However, certain species, like *N. beijingensis*, tend to target healthy individuals [13]. A recent study of 40 patients revealed that only 12.5% had no identifiable underlying disease [2]. It is worth noting that nocardiosis can occasionally affect individuals with sound immune systems, particularly those with pre-existing pulmonary disease, chronic obstructive pulmonary disease, bronchiectasis, asthma, and prior tuberculosis [6]. The infection is more commonly reported in males, with a female-to-male ratio of 1:3 [8,3]. In some cases, simultaneous infections with *Nocardia* and tuberculosis have been observed and are more likely to occur in HIV-infected patients [1].

The clinical presentation of pulmonary nocardiosis is non-specific [2]. Prominent symptoms are productive cough, chest pain, and rarely hemoptysis [4,6]. Most patients experience a subacute to chronic form of infection [2], with additional symptoms such as fever, weight loss, and malaise similar to mycobacterial disease [14]. The time taken from identifying symptoms to make the diagnosis can vary from three days to 42 days [2,4]. When imaging is conducted, it often reveals lesions in the superior lobe. These lesions can be mistaken for tuberculosis, mycotic infection (*Aspergillus*), or malignancy [5,2,8]. *Nocardia* usually results in poorly contained pneumonia, which causes necrosis, and sometimes erodes into adjacent bony structures, resulting in the misdiagnosis of malignancy [8]. Chest CT findings typically show focal areas of consolidation, macroscopic nodules, masses, or cavities [6].

Identifying *Nocardia* can be challenging as it can take days to weeks to grow in culture. It typically requires a minimum of 48-72 hours before colonies become visible [14]. It is important to observe the colonial morphology both macroscopically and microscopically for the presence of aerial hyphae. Gram stain usually shows beaded, branching gram-positive filaments that are partially acid-fast. Direct microscopy may also reveal hyphae-like branching [6]. The colony surface resembles "cotton candy" [14]. Some also recommend using the modified Kinyoun acid-fast stain [14]. Molecular techniques, such as polymerase chain reaction, restriction enzyme analysis, and 16s RNA gene sequencing (the gold standard for definitive identification of the species) [15], have revolutionized the identification of *Nocardia* species [8,16]. Recent reports have shown that matrix-assisted laser desorption ionization time-of-flight mass spectrometry (MALDI-TOF MS) is a rapid and simple technique for identifying *Nocardia* species [16]. Histology may show acute, chronic, or granulomatous changes [6]. It is important to note that caution should be exercised when interpreting the identification of *Nocardia* from the respiratory tract in a person without apparent pulmonary infection [7].

When it comes to treating nocardiosis, the preferred medication is TMP-SMX [1]. However, there is already 2% resistance to this drug, according to the information from the Centers for Disease Control and Prevention among 765 *Nocardia* samples submitted between 1995 and 2004 [4]. In such cases, alternative options include linezolid, carbapenems, and fourth-generation cephalosporins like ceftriaxone, cefepime, and amoxicillin-clavulanate [9,6]. Regarding the carbapenems, meropenem has been used successfully in the treatment of nocardiosis, though usually in combination with other antimicrobials [6]. According to some authors, imipenem is said to be more effective against most *Nocardia* species. A combination therapy of imipenem and cefotaxime, amikacin and TMP-SMX, imipenem and TMP-SMX, amikacin and cefotaxime, or amikacin and imipenem may improve efficacy [7]. Immunocompetent patients with pulmonary nocardiosis should undergo treatment for six to 12 months [7], while cutaneous nocardiosis should be treated for one to three months and a minimum of 12 months for immunocompromised patients or those with central nervous system infection [1,8,15].

The recurrence rate in patients with nocardiosis is 5% [17]. According to studies, patients with disseminated disease, compromised immune systems, advanced age, central nervous system involvement, and concurrent infections have a higher mortality rate [2]. Research has demonstrated mortality rates of 38.7%, which can increase to 64% in cases of disseminated nocardiosis and reach 100% in instances of central nervous system infection [8].

## Conclusions

The clinical presentation of nocardiosis, particularly pulmonary nocardiosis, can be non-specific, with symptoms such as productive cough, chest pain, and rarely hemoptysis. However, these symptoms can overlap with other conditions like tuberculosis, mycotic infections, or malignancy, making the diagnosis challenging. Timely and accurate diagnosis is crucial but can be challenging as *Nocardia* can take days to weeks to grow in culture and molecular techniques such as polymerase chain reaction and gene sequencing are often required for definitive identification, which at times need to be done at reference laboratories. It is worth noting that even immunocompetent individuals, particularly those with pre-existing pulmonary diseases, can be affected. The prognosis of nocardiosis can vary depending on various factors, in some cases with extremely high mortality rates. Lastly, even though uncommon, nocardiosis can recur and should be in the differential in a patient with persistent cavitation. 
